# CRISPR-TE: a web-based tool to generate single guide RNAs targeting transposable elements

**DOI:** 10.1186/s13100-024-00313-0

**Published:** 2024-02-01

**Authors:** Yixin Guo, Ziwei Xue, Meiting Gong, Siqian Jin, Xindi Wu, Wanlu Liu

**Affiliations:** 1 Department of Orthopedic Surgery of the Second Affiliated Hospital, and Centre of Biomedical Systems and Informatics of Zhejiang University-University of Edinburgh Institute (ZJU-UoE Institute), Zhejiang University School of Medicine, Zhejiang University, Zhejiang, Hangzhou 310003 China; 2 Future Health Laboratory, Innovation Center of Yangtze River Delta, Zhejiang University, Jiaxing, 314100 China

## Abstract

**Background:**

The CRISPR/Cas systems have emerged as powerful tools in genome engineering. Recent studies highlighting the crucial role of transposable elements (TEs) have stimulated research interest in manipulating these elements to understand their functions. However, designing single guide RNAs (sgRNAs) that are specific and efficient for TE manipulation is a significant challenge, given their sequence repetitiveness and high copy numbers. While various sgRNA design tools have been developed for gene editing, an optimized sgRNA designer for TE manipulation has yet to be established.

**Results:**

We present CRISPR-TE, a web-based application featuring an accessible graphical user interface, available at https://www.crisprte.cn/, and currently tailored to the human and mouse genomes. CRISPR-TE identifies all potential sgRNAs for TEs and provides a comprehensive solution for efficient TE targeting at both the single copy and subfamily levels. Our analysis shows that sgRNAs targeting TEs can more effectively target evolutionarily young TEs with conserved sequences at the subfamily level.

**Conclusions:**

CRISPR-TE offers a versatile framework for designing sgRNAs for TE targeting. CRISPR-TE is publicly accessible at https://www.crisprte.cn/ as an online web service and the source code of CRISPR-TE is available at https://github.com/WanluLiuLab/CRISPRTE/.

**Supplementary Information:**

The online version contains supplementary material available at 10.1186/s13100-024-00313-0.

## Introduction

Since the initial discovery of the CRISPR/Cas9 system for genome editing [1, 2], the development of catalytically inactive Cas9 variants has further facilitated its application in targeted gene expression activation [3, 4], repression [3], and base editing [5]. Transposable elements (TEs) are mobile DNA sequences capable of moving within the genome [6]. Though once deemed “genomic dark matter”, recent studies have suggested that TEs may act as cis-regulatory elements, contributing to gene regulation by serving as promoters, enhancers, silencers, and boundary elements [7]. For instance, in mouse early embryogenesis, the endogenous retrovirus MuERV-L serves as an alternative promoter for certain genes specific to the two-cell stage that are bound and induced by the transcription factor Dux [8]. In humans, the evolutionarily young transposable elements such as LTR7Y, LTR7, and LTR5HS harbor binding sites for several key transcription factors and are posited to regulate both human naïve pluripotency and germline lineage commitment [9–13].

The study of TE functions is challenging due to their high copy numbers and their sequence repetitiveness [7]. Consequently, designing sgRNAs that efficiently recruit CRISPR/Cas9 system to TEs is key for functionally probing their biological roles. Researchers have targeted individual TE copies with CRISPR/Cas9 or CRISPR inhibition (CRISPRi) systems to delete, insert, or repress specific copies, thereby studying their biological functions [14–18]. Moreover, there have been efforts to elucidate the functions of TE subfamilies using CRISPRi or CRISPR activation (CRISPRa), involving the design of sgRNAs that target multiple copies within certain TE subfamilies [11, 17, 19, 20]. However, these attempts to manipulate TE expression via CRISPRi or CRISPRa have largely relied on sgRNAs selected using gene-centric tools and on the manual design of sgRNAs targeting consensus sequences of TE copies. Employing similar strategies, we have used CRISPRi/a to silence or activate specific TE subfamilies, assessing their potential enhancer roles in human embryonic stem cells and primordial germ cells [12, 13]. Nonetheless, the prevailing CRISPR design tools are primarily gene-centric and fail to provide adequate on- or off-target information for TEs, limiting in-depth TE functional studies.

In this study, we introduce CRISPR-TE, a web-based bioinformatics tool specifically for designing CRISPR/Cas sgRNAs targeting transposable elements. Our tool can design sgRNAs to target individual TE copies or combinations of sgRNAs to target TE subfamilies. Moreover, CRISPR-TE provides an interactive web interface with swift query capabilities, enabling convenient access and analysis of detailed sgRNA information for researchers. In summary, CRISPR-TE represents a valuable resource for researchers investigating the role of TEs in the genome, facilitating more comprehensive and precise studies of these repetitive elements.

## Results

### CRISPR-TE workflow

**Fig. 1:**
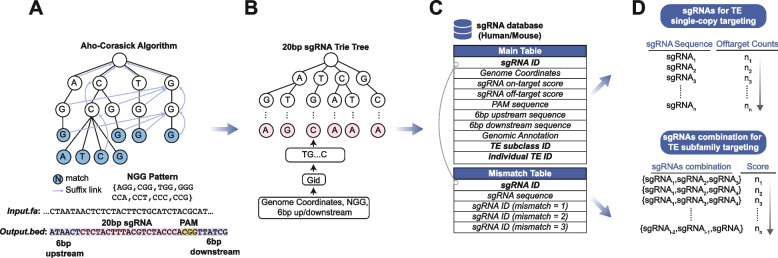
CRISPR-TE Workflow for Designing sgRNAs Targeting Transposable Elements. **A** Efficiently searches for all potential sgRNA target sites (N20NGG) using the Aho-Corasick algorithm on the genome FASTA file. **B** Employs a trie data structure to efficiently store sgRNA sequences, facilitating hamming distance mismatch searches. **C** Stores comprehensive sgRNA information for human and mouse genomes in a PostgreSQL database, consisting of two tables: the main table contains sgRNA sequences and coordinates, 6bp upstream and downstream sequences, on/off-target scores, genetic element classes, and the targeted TE (if applicable). **D** CRISPR-TE provides two approaches for sgRNA design targeting TEs: (i) targeting a single copy with minimal off-targets, and (ii) targeting a TE subfamily using optimal sgRNA combinations determined by a greedy algorithm

CRISPR-TE first constructs a database of sgRNAs by scanning human and mouse genomes for potential target sites containing the PAM (protospacer adjacent motif) sequence (5′-NGG-3′ for SpCas9 from *S. pyogenes*). Upon input of a genome file, the Aho-Corasick pattern matching algorithm efficiently identifies all N20NGG patterns within the reference genome [21] (Fig. 1A). A retrieval tree (trie) data structure stores all sgRNAs, their genomic locations, and 6 bp downstream and upstream sequences (Fig. 1B). This data structure enables efficient computation of sequence mismatch neighborhoods. Additional data, including sgRNA on-target activity efficiency [22, 23], TE-specified off-target scores, TE subfamily, individual TE ID (if any), and overlapping genetic elements such as exons, introns, promoter-TSS, intergenic regions, are calculated and stored in the main database table managed by PostgreSQL. Queries for individual TE ID and their genomic coordinates are also available on the CRISPR-TE website. The sgRNA ID (gid) acts as a foreign key linking the database mismatch table, which contains gid, sgRNA sequence, and mismatch neighborhoods (Fig. 1C). CRISPR-TE offers two strategies for TE-specific sgRNA design: 1) targeting a single individual TE copy with minimal off-targets or 2) targeting TE subfamilies using optimized sgRNA combinations ranked by a greedy algorithm (Fig. 1D). These strategies provide researchers with comprehensive options for TE studies, enabling them to select the approach best suited to their experimental goals.

### Web interface

**Fig. 2:**
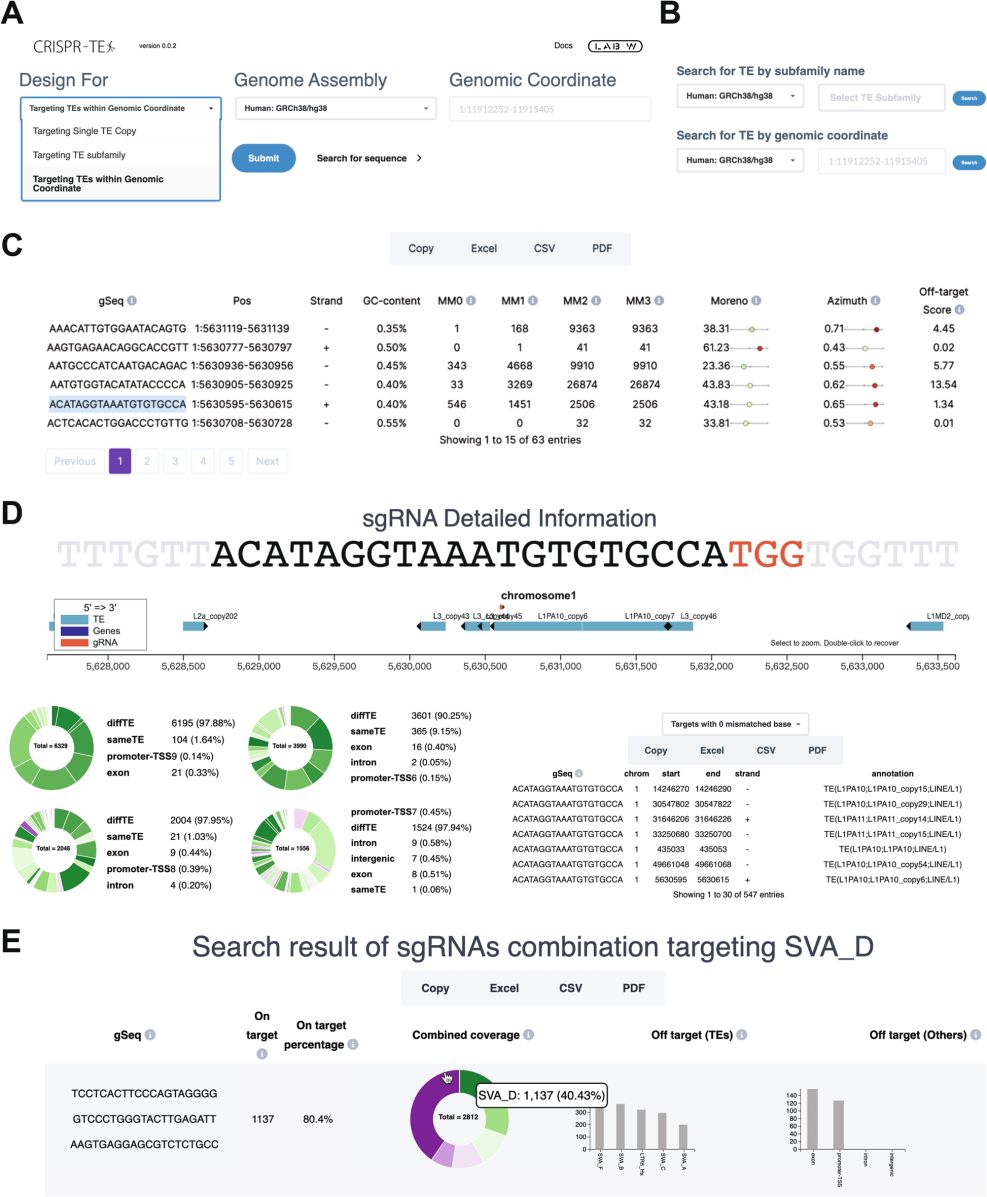
Screenshot of The CRISPR-TE Web Tool Interface. **A** The CRISPR-TE homepage, which requires three types of input: (i) Design purpose, (ii) Genome assembly, and (iii) Target TE copy ID or genomic coordinates. **B** Tool is provided for querying individual TE copy IDs and their genomic coordinates. **C** After submitting, CRISPR-TE displays all possible sgRNAs along with detailed information, including sgRNA sequence, coordinates, GC content, mismatches, on-target score, and off-target score. **D** CRISPR-TE enables users to examine the locations of sgRNAs on the genome, alongside other genomic features, by clicking on individual sgRNAs. The pie chart on the left illustrates the proportions of target sites with various mismatch counts. A list of all off-target sgRNA, including their sequences, genomic coordinates, and associated genetic element classes, is shown on the right. **E** The results for sgRNA combinations targeting TE subfamilies are presented. This includes the sgRNA sequences, the number of on-target sites, the on-target percentage for the queried TE subfamily, the sgRNA combination coverage, and off-targets on TEs and other genetic element classes

CRISPR-TE features a user-friendly web interface for designing sgRNAs targeting transposable elements. Users input the design objective (targeting TE single copies or subfamilies), the genome assembly (human or mouse), and the name of the targeted TE subfamily or individual TE ID (Fig. 2A). The annotation query function on the CRISPR-TE website allows users to search for specific genomic coordinate or individual TE IDs (Fig. 2B). After submitting the sgRNAs design, CRISPR-TE generates an interactive table displaying sgRNA sequences, coordinates, potential off-target numbers with 0, 1, 2, or 3 mismatches, and on/off-target activity scores (Fig. 2C). Detailed sgRNA information becomes accessible to users by clicking on each row in the summary table. A color-coded graphical representation of the sgRNA target site help users inspect candidate sgRNAs based on their locations (Fig. 2D). Summary pie charts depict the proportions of target sites by mismatch number and lists of off-target sgRNAs with their sequences and target sites further aid in the selection of suitable sgRNAs (Fig. 2D). For designing sgRNAs for TE subfamilies, CRISPR-TE generates combinations intended to maximize coverage of the queried TE subfamily. To balance coverage and computational complexity, CRISPR-TE currently supports designing combinations of three sgRNAs. Pie charts and bar plots visualize the proportion of on-target sites and the number of off-target sites for each sgRNA (Fig. 2E). Users can download the results in Excel, CSV, and PDF format for further analysis and documentation.

### TE sgRNA analysis of human and mouse

**Fig. 3:**
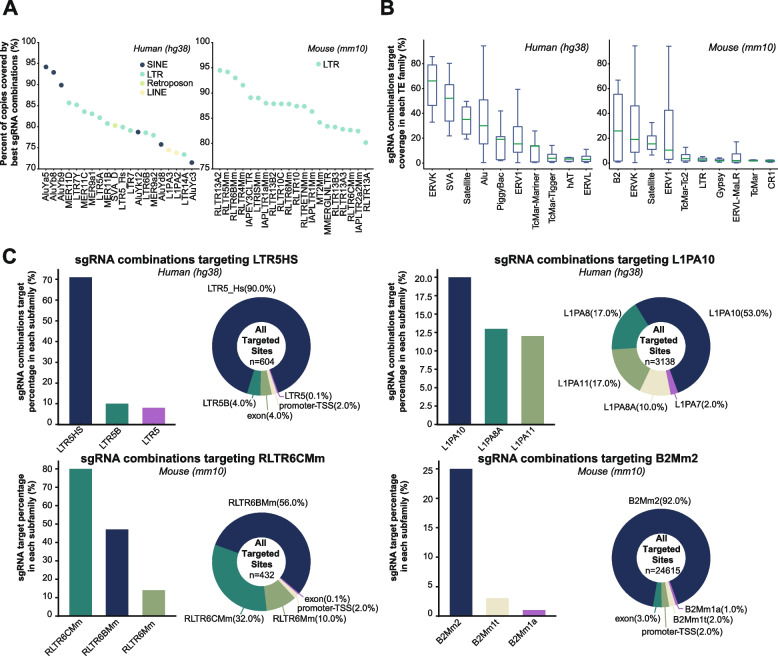
Analysis of TE sgRNAs in Human and Mouse. **A** Displays the top 20 TE subfamilies with the highest coverage using the best three sgRNA combinations in human (left panel) and mouse (right panel). **B** Box plots showing the coverage achieved by the best three sgRNA combinations in each TE family for human (left panel) and mouse (right panel). **C**. Examples of sgRNAs designed by CRISPR-TE for targeting LTR5HS (upper left panel), L1PA10 (upper right panel), RLTR6BMm (bottom left panel), and B2Mm2 (bottom right panel) TEs. The bar plots indicate the targeted percentage of copies for the top three TE subfamilies using the best three sgRNA combinations. The pie charts represent the genomic distribution of all targeted sites by the corresponding best three sgRNA combinations

As TEs integrate into the genome, their sequences diverge due to the accumulation of random mutations and truncations. Evolutionarily young subfamilies, often considered as currently or recently active, possess highly similar sequences across different copies. In contrast, sequences of evolutionarily old subfamilies typically exhibit a greater degree of divergence from their consensus sequences [24]. We analyzed the percentage copies covered by three sgRNA combinations for all TE subfamilies. As anticipated, sgRNA combinations designed by CRISPR-TE target evolutionarily young families such as LTR7Y, LTR5HS, SVA-D in humans with higher coverage compared to older families (Fig. 3A and Fig. S1). Specifically, in humans, young TE subfamilies like ERVK and SVA show over 50% coverage with three sgRNAs. Similarly, in mice, B2 and ERVK rank as the top covered TE subfamilies (Fig. 3B). Furthermore, we discovered that evolutionarily young TEs in human and mouse, such as LTR5HS (coverage ranked at 15 in human TE subfamilies) and RLTR6CMm (coverage ranked at 22 in mouse TE subfamilies), can be targeted with over 70% coverage using CRISPR-TE-designed sgRNA combinations at the subfamily level, despite the possibility that some sgRNAs may also target other TE subfamilies with similar sequences (Fig. 3C). Conversely, for other relatively older TEs such as L1PA10  (coverage ranked at 223 for human TE subfamilies) and B2Mm2 (coverage ranked at 201 for mouse TE subfamilies), CRISPR-TE-designed sgRNA combinations can target only about 20% of copies, although the majority of the designed sgRNAs accurately target the intended TEs (Fig. 3C). In conclusion, the effectiveness of sgRNA targeting by CRISPR-TE is strongly correlated with the age of the TE, with younger TEs being more amenable to efficient targeting.

## Discussion

This study introduces CRISPR-TE, a specialized sgRNA design tool tailored for the unique challenges associated with TE targeting in genome editing. Our novel approach for sgRNA design offers a significant advancement over traditional gene-targeting tools, addressing the high copy number and sequence repetitiveness that have long hindered effective TE manipulation. Our results indicate that CRISPR-TE can accurately target TE subfamilies, particularly those that are evolutionarily young and exhibit conserved sequences. The tool’s ability to target these TEs with higher coverage suggests that CRISPR-TE is adept at identifying and leveraging the less divergent sequences within these younger subfamilies. This is a crucial development, as it facilitates the functional analysis of TEs that may play significant roles in gene regulation and genome architecture.

While CRISPR-TE has shown promising results, we recognize certain limitations in its current iteration. At present, CRISPR-TE is tailored only to human and mouse genomes using SpCas9. Given the ubiquitous presence and regulatory significance of TEs in various plant species, including maize, where they play a pivotal role in phenotypic regulation [25], future enhancements will aim to broaden the tool’s species compatibility and include additional Cas enzymes like Cas12 or Cas13 orthologues [26]. We are committed to extending CRISPR-TE’s functionality to encompass a wider array of species and Cas variants in our subsequent updates.

The current version supports three sgRNA combinations for targeting TE subfamilies, primarily to manage the computational complexity, which grows exponentially with additional sgRNAs. This limitation may restrict the tool’s effectiveness, particularly when addressing evolutionarily older TE subfamilies that require more comprehensive sgRNA coverage. To overcome this, future development will focus on refining our greedy algorithm to allow for an increased number of sgRNA combinations, which could enhance the scope of TE subfamily targeting. However, the experimental delivery of multiple sgRNAs into cellular systems or animal models poses its own set of challenges [27], particularly when investigating TE subfamily functions.

Furthermore, the potential for off-target effects is an inherent concern due to the repetitive nature of TE sequences. Although CRISPR-TE includes an on-target and off-target scoring system, these algorithms were originally developed for gene targeting and may not be fully optimized for TEs [28]. Advances in the specificity of on-target and off-target predictions for TEs remain a priority for future refinement. The incorporation of machine learning algorithms is anticipated to improve the precision of sgRNA efficacy predictions, thereby mitigating the risk of off-target effects [29, 30].

In conclusion, CRISPR-TE represents a notable step forward in the field of genome engineering, allowing researchers to explore the possible functions of TEs using genome editing tools. As we further imrpove this tool, we anticipate it to become an essential resource for TE research, providing deeper insights in understanding the repetitive elements in the genome.

## Methods

### sgRNA sequence search, annotation, and storage

We utilized the Aho-Corasick string matching algorithm to screen the genome sequences for all occurrences of the N20NGG pattern on both positive and negative strands. We then saved all potential sgRNA sequences into a modified Trie tree data structure. These sequences were classified based on various genetic elements, such as exons, introns, promoter-TSS, and intergenic regions. The genome assembly and annotation versions used were GRCh38.97 for human (http://www.ensembl.org/Homo_sapiens/) and GRCm38.97 for mouse (https://www.ensembl.org/Mus_musculus/). We obtained annotations for transposable element (TE) subfamilies and individual TE IDs from RepeatMasker (https://www.repeatmasker.org/). Using Trie tree structure, we performed mismatched string pattern matching to identify N20NGG sequences with fewer than 3 mismatched nucleotides in the genome for each sgRNA. We stored the resulting nucleotide sequences, their genomic coordinates, annotations, and mismatch information in a PostgreSQL (version 14.3) database for efficient indexing and rapid searching.

### sgRNA combination search and off-target score

We proposed a computation time-optimized greedy search algorithm to identify all potential sgRNA combinations that can cover most copies of a TE subfamily while ensuring a minimum number of off-target sites in other genetic elements. We ranked all sgRNAs targeting any copies of each TE subfamily based on their total coverage of copies. The sgRNA combination score was computed as a weighted sum of coverage and off-target events, defined by:

$$SCORE\;=\;Coverage-\;\lambda_1\;\times\;W_1\;-\lambda_2\;\times\;\left(\lambda_3\;\times\;W_2\;+\;\lambda_4\;\times\;W_3\;+\;\lambda_5\;\times W_4\;+\;\lambda_6\;\times W_5\right)$$

where coverage is the percentage of TE subfamily copies covered by the current sgRNA combination, *W*_1_ is the number of off-target TEs, *W*_2_ is the number of off-targets to promoter-TSS, *W*_3_ is the number of off-target exons, *W*_4_ is the number of off-target introns, *W*_5_ is the number of off-target intergenic regions, with weights λ_1_ = 1e-3, λ_2_ = 1e-4, λ_3_ = 0.4, λ_4_ = 0.3, λ_5_ = 0.4, λ_6_ = 0.3, set as default parameters.

We employed a greedy search strategy that involved selecting the top n sgRNAs with the highest combination scores and subsequently ranking the remaining sgRNAs by the increment of the combination score. We greedily added the most optimal sgRNAs to the current combination at each iteration to obtain the final combination of sgRNAs for targeting copies of a TE subfamily.

### Implementation of the CRISPR-TE web server

We developed a web server that enables users to search for sgRNAs targeting TEs, using an intuitive and user-friendly data browser. The front-end interface of the web server was created with HTML5 and CSS3, and all data visualizations were produced using the D3.js framework [31]. The back-end data, containing sgRNA sequences and annotations, was managed by the PostgreSQL database system, facilitating prompt responses to user queries. Python3 (v3.9.12) and Django (v3.2.5) were used for communication between the front-end and back-end. The website is accessible at https://www.crisprte.cn/ without the need for registration or login. The CRISPR-TE website’s functionality was thoroughly tested on Google Chrome and Apple Safari browsers. The site is deployed on an Nginx web server (v1.18.0) running on a Linux Ubuntu (v20.04.5 LTS) cloud server system.

### Supplementary Information

**Additional file 1: Figure S1.** sgRNA Combinations Targeting TE Subfamilies in Human and Mouse. All TE subfamilies are ranked by the targeted coverage using the best sgRNA combinations for human (left panel) and mouse (right panel).

## Data Availability

The CRISPR-TE web tool is publicly available at https://www.crisprte.cn/. The source code of CRISPR-TE is accessible at https://github.com/WanluLiuLab/CRISPRTE/.
