# Supplementary material for: CRISPR-TE: a web-based tool to generate single guide RNAs targeting transposable elements
Source: Mob DNA. 2024 Feb 1;15:3. doi: 10.1186/s13100-024-00313-0 (PMC10832116; doi:10.1186/s13100-024-00313-0)
Supplement: Supplementary file 1 — Additional file 1: Figure S1. sgRNA Combinations Targeting TE Subfamilies in Human and Mouse. All TE subfamilies are ranked by the targeted coverage using the best sgRNA combinations for human (left panel) and mouse (right panel). [file 13100_2024_313_MOESM1_ESM.pdf]

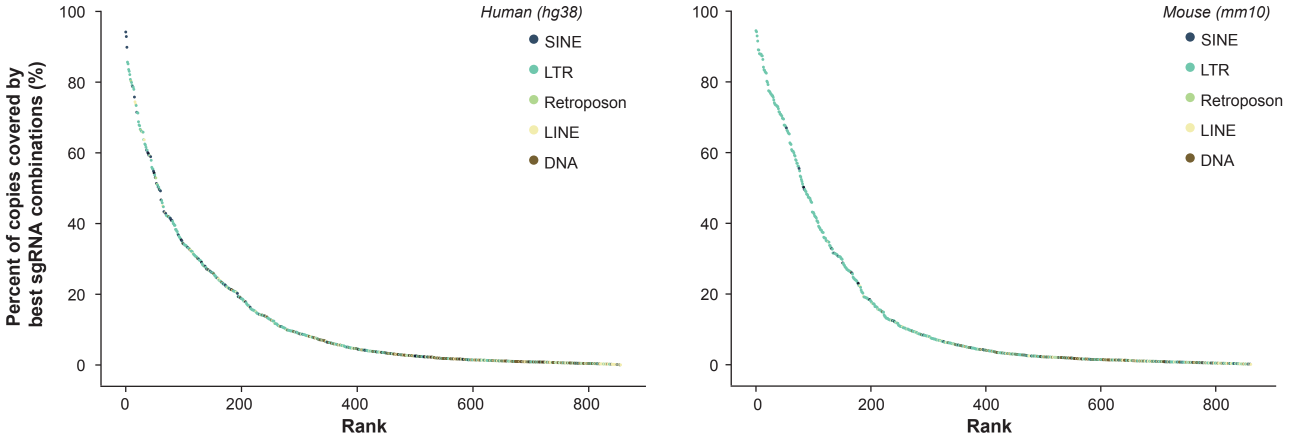

Figure S1. sgRNA Combinations Targeting TE Subfamilies in Human and Mouse. All TE subfamilies are ranked by the targeted coverage using the best sgRNA combinations for human (left panel) and mouse (right panel).
